# Allele and dosage specificity of the *Peg3* imprinted domain

**DOI:** 10.1371/journal.pone.0197069

**Published:** 2018-05-07

**Authors:** Corey L. Bretz, Wesley D. Frey, Ryoichi Teruyama, Joomyeong Kim

**Affiliations:** Department of Biological Sciences, Louisiana State University, Baton Rouge, LA, United States of America; University of Bristol, UNITED KINGDOM

## Abstract

The biological impetus for gene dosage and allele specificity of mammalian imprinted genes is not fully understood. To address this, we generated and analyzed four sets of mice from a single breeding scheme with varying allelic expression and gene dosage of the *Peg3* domain. The mutants with abrogation of the two paternally expressed genes, *Peg3* and *Usp29*, showed a significant decrease in growth rates for both males and females, while the mutants with biallelic expression of *Peg3* and *Usp29* resulted in an increased growth rate of female mice only. The mutant cohort with biallelic expression of *Peg3* and *Usp29* tended to have greater numbers of pups compared to the other genotypes. The mutants with switched active alleles displayed overall similar phenotypes to the wild type, but did show some differences in gene expression, suggesting potential non-redundant roles contributed by the maternal and paternal alleles. Overall, this study demonstrates a novel *in vivo* approach to investigate the allele and dosage specificity of mammalian imprinted domains.

## Introduction

In placental mammals, the majority of autosomal genes have two functional copies, which are contributed by two parents. In contrast, a very small subset of genes has only one functional copy due to genomic imprinting, by which one allele is inactivated by epigenetic mechanisms [1–3]. This mono-allelic expression or single gene dosage driven by genomic imprinting has been functionally selected and preserved during the evolution of eutherian mammals [1–3]. Each imprinted gene is also expressed from one specified allele, either the maternally or paternally inherited copy, and this allele specificity is well conserved among all the eutherian mammals. About 100 to 200 genes are imprinted in mammalian genomes, and these genes are usually expressed in early-stage embryo, placenta and brain [1–3]. Mutations in these imprinted genes have very similar functional outcomes, such as changes in fetal growth rates and perturbations in animal nurturing behaviors. Yet, genomic imprinting is found only within the eutherian lineage that has a very unusual reproduction strategy involving viviparity and placentation. Thus, genomic imprinting is thought to have co-evolved with the reproduction scheme of placental mammals to control the dosage of the genes that play critical roles in the reproduction-related physiology and behaviors [4–7]. Nevertheless, the actual biological reasons for the allele and dosage specificity associated with each imprinted gene have not been well understood so far.

*Peg3* (Paternally expressed gene 3) is the founding member of the 500-kb imprinted domain localized in human chromosome 19q13.4/proximal mouse chromosome 7 [8–10]. This domain contains paternally expressed *Peg3*, *Usp29*, *Zfp264*, *APeg3* and maternally expressed *Zim1*, *Zim2*, *Zim3* [11]. As described earlier, the allele and dosage specificity of the *Peg3* domain are well conserved among all the placental mammals. The functions of these genes are also closely associated with controlling fetal growth rates and nurturing behaviors [12–15]. The imprinting of this domain is controlled through an ICR (Imprinting Control Region), the Peg3-DMR (Differentially Methylated Region), which encompasses the bidirectional promoter for *Peg3*/*Usp29* [16, 17]. Deletion of this ICR abolishes the transcription of *Peg3* and *Usp29*, and also causes biallelic expression, or double dosage for some of the adjacent imprinted genes [18]. In particular, the maternally expressed *Zim1* becomes biallelic due to the reactivation of the paternal allele. The Peg3-DMR obtains its gametic DNA methylation during oogenesis, which is then maintained in somatic cells throughout the lifetime of the animal [18, 19]. Recent studies further revealed that one upstream alternative promoter, termed U1, is responsible for establishing this oocyte-specific DNA methylation on the Peg3-DMR [20]. Deletion of this alternative promoter results in a loss of the allele-specific methylation on the Peg3-DMR, subsequently causing biallelic expression or double dosage of *Peg3* and *Usp29*.

In the current study, we sought to characterize the allele and dosage specificity associated with the *Peg3* domain by using two mutant alleles that target the Peg3-DMR and the alternative U1 promoter. According to the results, the gene dosage of the *Peg3* domain is indeed closely associated with the growth rates of the animals. Also, although the allele switching of the *Peg3* domain did not cause any major impact on animal growth, detailed examinations suggest the presence of potential differences in gene expression between the two alleles.

## Results

### Generation of four genotypes with different dosage and allele specificity

**Fig 1:**
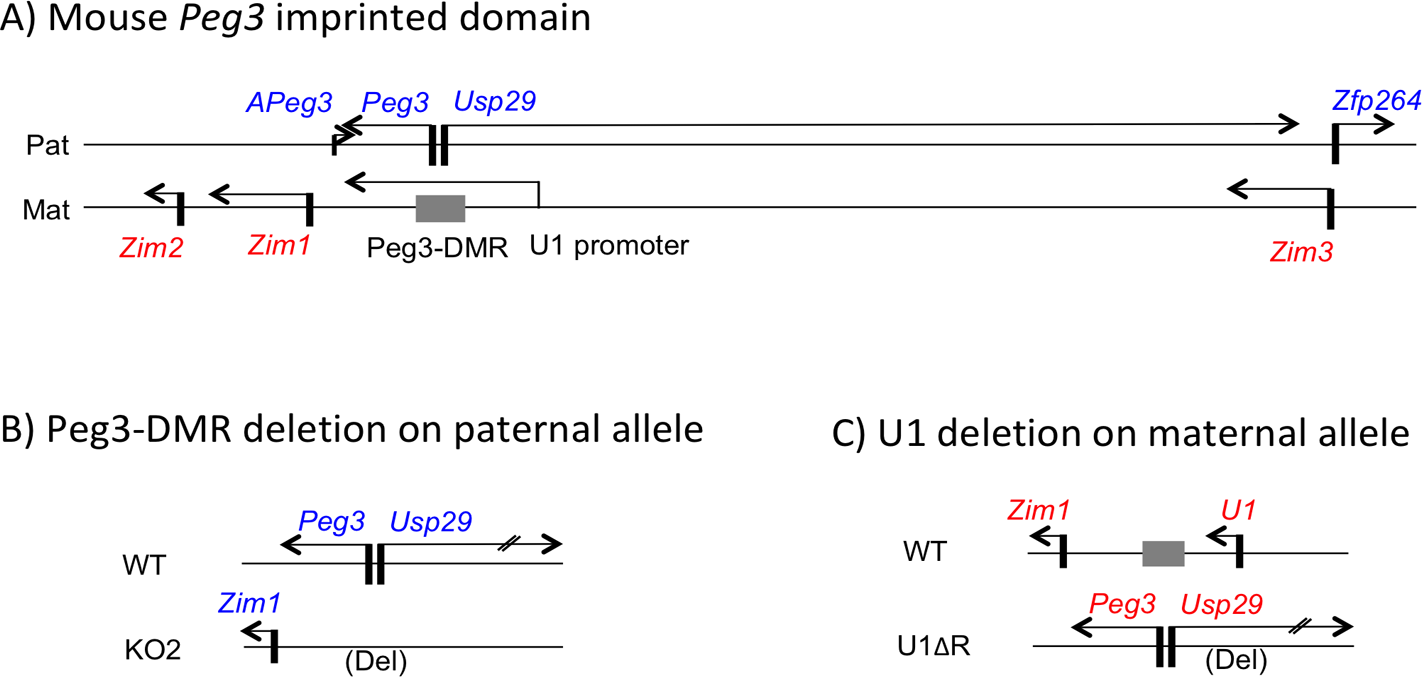
Schematic representation of the mouse *Peg3* domain and two mutant alleles. (**A**) Mouse *Peg3* imprinted domain. The 500-kb imprinted domain contains 7 genes, which are indicated by arrows. Paternally expressed *Peg3*, *Usp29*, *Zfp264*, *APeg3* are indicated by the corresponding gene names with blue font, whereas the maternally expressed *Zim1*, *Zim2*, *Zim3* are indicated by the corresponding gene names with red font. The 4-kb Peg3-DMR controls the imprinting of the entire domain, which is indicated by a grey box (nucleotide positions 6,718,442–6,733,840 in the mouse chromosome 7 of mm10). In contrast, the alternative U1 promoter is known to target *de novo* DNA methylation to the maternal allele of the Peg3-DMR. This alternative promoter is localized 20-kb upstream of the Peg3-DMR, indicated with a small arrow (nucleotide positions 6,750,937–6,751,933 in the mouse chromosome 7 of mm10). (**B**) Deletion of the paternal allele of the Peg3-DMR abolishes the transcription of two paternally expressed genes, *Peg3* and *Usp29*, and consequently causes the reactivation of the paternal *Zim1*. (**C**) Deletion of the U1 promoter during oogenesis results in the reactivation of two paternally expressed genes, *Peg3* and *Usp29*, from the maternal allele, and subsequently causes significant down-regulation of the maternal *Zim1*.

In the current study, we used two deletion alleles to generate the mutant pups with different dosage and allele specificity. The first mutant allele termed KO2 is deigned to delete the Peg3-DMR, a 4-kb genomic region encompassing the bidirectional promoter for the two paternally expressed genes, *Peg3* and *Usp29* (**Fig 1B**). Paternal deletion of this 4-kb region abolishes the expression of *Peg3* and *Usp29*, and also causes reactivation of the maternally expressed *Zim1* from the paternal allele [17]. The second mutant allele termed U1ΔR is designed to delete the 1-kb genomic region encompassing the alternative U1 promoter, which is located 20-kb upstream of the Peg3-DMR (**Fig 1A**). The transcription driven by this alternative promoter during oogenesis is responsible for oocyte-specific DNA methylation on the Peg3-DMR. Thus, maternal deletion of the U1 promoter results in the loss of oocyte-driven DNA methylation on the Peg3-DMR, and subsequent reactivation of the two paternally expressed genes, *Peg3* and *Usp29*, from the maternal allele (**Fig 1C**). As a consequence, the expression of *Zim1* is also significantly down-regulated to its basal levels from the maternal allele, exhibiting about 3 to 5% of the normal expression levels [20].

**Fig 2:**
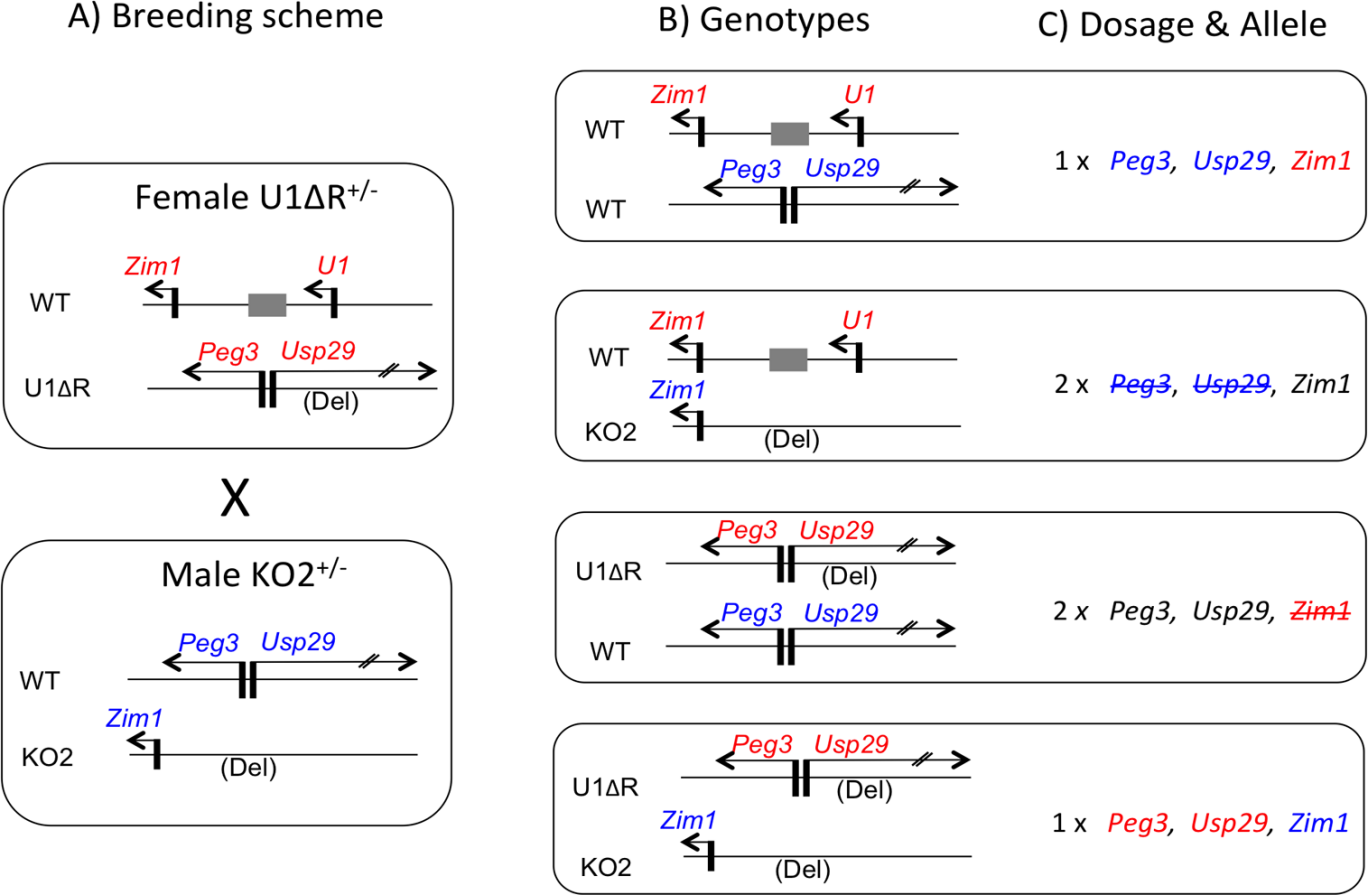
Breeding scheme and the allele and dosage specificity of the subsequent progeny. (**A**) Schematic representation of two deletion mutants for the breeding. The females carry the deletion allele for the alternative U1 promoter (U1ΔR), whereas the males carry the deletion allele for the Peg3-DMR (KO2). (**B**) The intercrossing of these two mutants produces the pups with 4 different genotypes: WT/WT, WT/KO2, U1ΔR/WT, U1ΔR/KO2. (**C**) Given the known effects by two mutant alleles, each genotype is predicted to have different dosage and allele specificity for the genes of the *Peg3* domain. These predicted outcomes are summarized in the following manner. The 1 X indicates the expression from a single allele, whereas the 2 X indicates the expression from two alleles. The genes with red font indicate the expression from the maternal allele, whereas those with blue font indicate the expression from the paternal allele. The genes with black indicate biallelic expression. The genes with strike-through indicate the significant down-regulation or no expression from that particular allele.

As a breeding scheme, we crossed 10 females that were heterozygous for the U1ΔR allele with 5 males that were heterozygous for the KO2 allele (**Fig 2A**). This breeding scheme with the two sets of heterozygotes was designed to derive the following four groups of pups with different genotypes (**Fig 2B**). The first group is the pups with two wild-type alleles, displaying normal allele and dosage specificity. In this genotype, referred to as simply 'WT' hereafter, *Peg3* and *Usp29* are expressed from the paternal allele whereas *Zim1* is expressed from the maternal allele (**Fig 2C**). The second group is the pups with the paternal transmission of the KO2 allele. In this genotype, referred to as 'KO2', the expression of *Peg3* and *Usp29* is abolished, and the expression of *Zim1* becomes biallelic due to the reactivation of the paternal *Zim1*. The third group is the pups with the maternal transmission of the U1ΔR allele. In this genotype, referred to as 'U1', the expression of *Peg3* and *Usp29* becomes biallelic due to the reactivation of the maternal allele, and also significant down-regulation of the maternal *Zim1*. Finally, the fourth group is the pups with the maternal and paternal transmission of U1ΔR and KO2, respectively. In this genotype, referred to as 'U1/KO2', *Peg3* and *Usp29* are expressed from the maternal allele, while *Zim1* is expressed from the paternal allele. We performed this series of breeding experiments, which successfully generated a total of 18 litters of pups with all of the predicted genotypes.

### DNA methylation of the *Peg3* domain among the four genotypes

**Fig 3:**
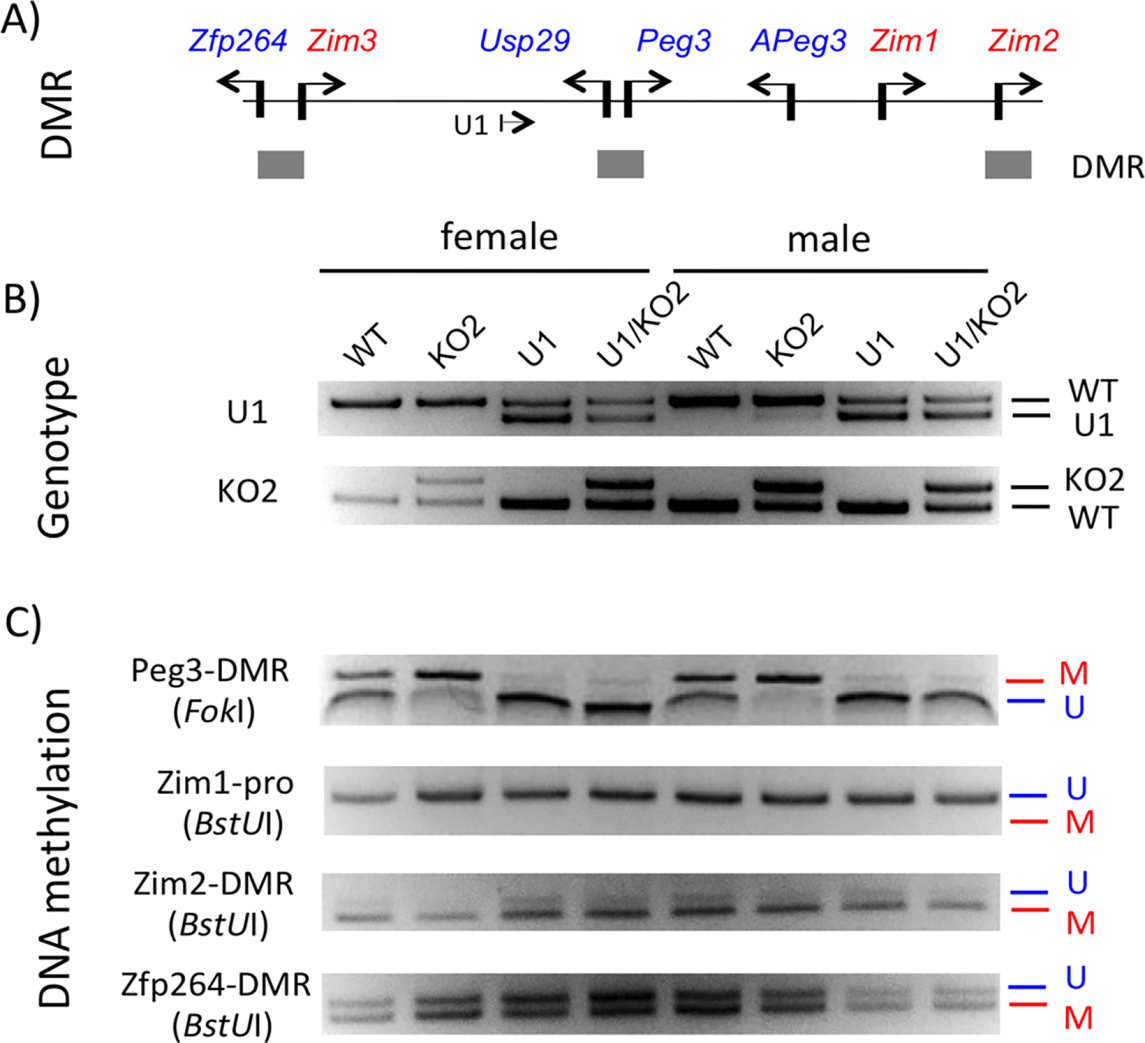
DNA methylation status of the *Peg3* domain among the four genotypes. (**A**) The positions of three DMRs are indicated on the genomic map of the *Peg3* domain. (**B**) The genotypes of the female and male sets of the pups are presented along with the results derived from the two sets of PCR amplifying the deleted regions. (**C**) The DNA from the four genotypes of both sexes were treated with bisulfite conversion protocol for DNA methylation analyses. The DNA were then amplified with four primer sets targeting the Peg3-DMR, the promoter of *Zim1*, Zim2-DMR and the Zfp264-DMR. The amplified products were digested with individual restriction enzymes that can differentiate the CpG methylation status of the original DNA, which are indicated with (U)nmethylation and (M)ethylation for a given CpG site.

The generated pups were analyzed in terms of their DNA methylation levels (**Fig 3**). This series of analyses used the DNA that had been isolated from the tails of the female and male sets. The isolated DNA were first genotyped with the primer sets targeting two deleted regions, the 1-kb genomic region harboring U1 and the 4-kb genomic region covering the Peg3-DMR (**Fig 3B**). The DNA were later treated with the bisulfite conversion protocol [21], which were then used for amplifying the following four target regions: Peg3-DMR, the promoter of *Zim1*, Zim2-DMR, and Zfp264-DMR (**Fig 3A**). The amplified DNA were subsequently analyzed with the COBRA (COmbined Bisulfite Restriction Analysis) protocol [22]. As shown in **Fig 3C**, the DMRs of the *Peg3* and *Zfp264* displayed about 50% DNA methylation levels in the WT samples (lane 1 and 5), while the promoter of *Zim1* did not show any levels of DNA methylation. In the case of the KO2 samples (lane 2 and 6), the Peg3-DMR showed a complete methylation pattern. This agrees with the fact that the amplified products were derived only from the methylated maternal allele, but not from the paternal allele due to the deletion of the entire 4-kb Peg3-DMR. In the case of the U1 samples (lane 3 and 7), the maternal allele of the Peg3-DMR was inherited as an unmethylated allele similar to the paternal allele, thus showing a complete unmethylation pattern. In the case of the U1/KO2 samples (lane 4 and 8), the remaining maternal allele of the Peg3-DMR showed a complete unmethylation pattern, which agrees with the fact that the U1 deletion causes a loss of DNA methylation on the remaining maternal allele. On the other hand, the DNA methylation patterns of the other DMRs and the promoter of *Zim1* were not affected at all by the two deletions. This is consistent with the previous observations from the two studies analyzing KO2 and U1 mutants individually [17, 20]. Taken together, this series of DNA methylation analyses confirmed that the generated pups with the four genotypes maintain the DNA methylation pattern as predicted.

### Expression levels of the imprinted genes in the four genotypes

**Fig 4:**
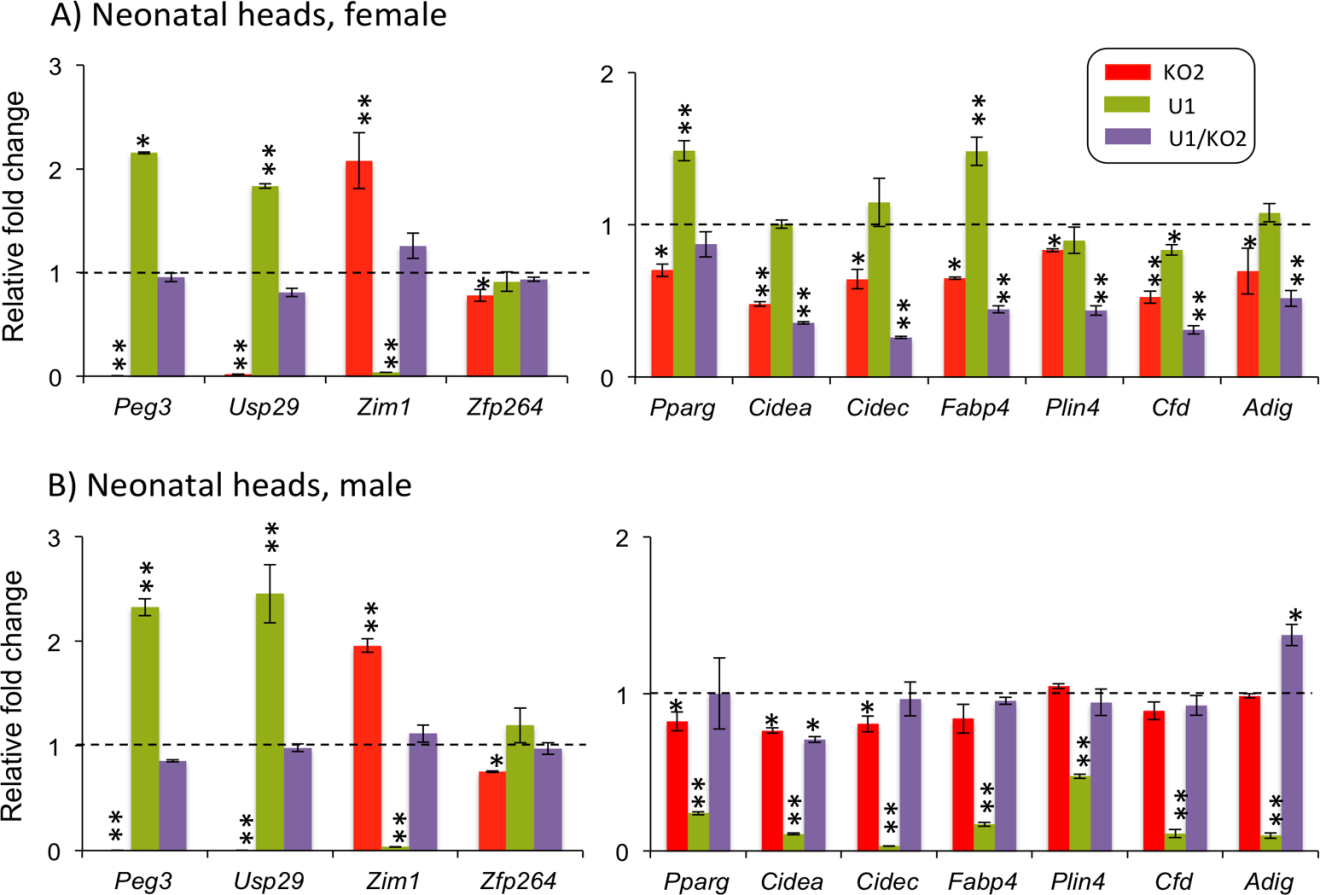
Expression levels of the *Peg3* domain and the gene set for lipid metabolism among the four genotypes. A series of qRT-PCR-based analyses were performed using three sets of total RNA isolated from the neonatal heads of the four genotypes of females (**A**) and males (**B**). The expression levels of the imprinted genes of the *Peg3* domain were summarized on the graph on left, whereas the expression levels of the gene set for lipid metabolism on the graph on right. The expression levels of each gene were first normalized with an internal control (β-actin). The subsequent value was further compared to that of the WT sample, which has been indicated with dotted lines. The relative levels were presented with standards deviation. Statistically significant differences in expression have been indicated with * being less than 0.05 and ** being less than 0.001. The corresponding p values were also pointed out in the text. This set of analyses was repeated using three biological replicates.

The generated pups were analyzed in terms of their expression levels of the individual genes within the *Peg3* domain. For this series of analyses, we isolated total RNA from the female (n = 3) and male (n = 3) sets of one-day-old neonatal heads representing the four genotypes (**Fig 4**). The isolated RNA was converted into cDNA, and subsequently used as templates for qRT-PCR analyses. This series of expression analyses focused mainly on the following four genes for the *Peg3* domain: *Peg3*, *Usp29*, *Zim1* and *Zfp264*. The other remaining genes, *Zim2*, *Zim3* and *APeg3*, were omitted due to their low expression levels in neonatal heads (**Fig 1A**). The results are summarized as follows. First, the KO2 samples showed no detectable levels of expression for both *Peg3* and *Usp29* (*p* = 0.0004, Mann-Whitney U test), yet exhibited a 2-fold up-regulation of *Zim1* (*p* = 0.0004, Mann-Whitney U test) compared to the levels observed from the WT samples (red bars in **Fig 4**). This was expected since the deletion of the Peg3-DMR in the KO2 samples abolishes the bidirectional promoter for *Peg3*/*Usp29*. The up-regulation of *Zim1* also has been observed from the various mutants targeting the *Peg3* locus [16, 17, 20], but the exact 2-fold up-regulation of *Zim1* appeared to be very precise given the predicted double gene dosage in the KO2 sample (**Fig 2C**). The overall expression patterns of genes in the *Peg3* domain from the KO2 samples were also similar between two sexes. Second, the U1 samples displayed 2-fold up-regulation for both *Peg3* and *Usp29* (*p* = 0.0004 and 0.0061 respectively, Mann-Whitney U test), and also significant down-regulation of *Zim1* (*p* = 0.0004, Mann-Whitney U test) to basal levels (green bars in **Fig 4**). The 2-fold up-regulation of *Peg3* and *Usp29* was consistent with the predicted double gene dosage for both genes, which was presumably caused by the reactivation of the maternal allele by the U1 deletion. The overall expression patterns of genes in the *Peg3* domain from the U1 samples were also similar between two sexes. Third, the U1/KO2 samples displayed similar levels of expression for the four genes as compared to those observed from the WT samples (purple bars in **Fig 4**). This suggests that the reactivated maternal allele of the Peg3-DMR *via* the U1 deletion may have rescued the abnormal expression patterns of the *Peg3* domain, which had been caused by the paternal deletion of the Peg3-DMR. It is, however, interesting to note that the expression levels of the *Peg3* domain were slightly different between WT and U1/KO2. Overall, this series of expression analyses confirmed that the predicted gene dosages of the individual genes were indeed manifested as the corresponding expression level differences between the individual genotypes.

### Expression levels of the other genes associated with *Peg3* in the four genotypes

We also tested the expression levels of several genes that are known to be closely associated with the gene dosage of *Peg3*. In particular, mutations in *Peg3* are known to affect the transcription levels of the two particular gene sets: the genes involved in lipid metabolism and several genes highly expressed in the hypothalamus [14]. Thus, we analyzed the expression levels of these genes using male (n = 3) and female (n = 3) cDNA sets derived from the four genotypes. First, we measured the expression levels of the following genes for lipid metabolism: *Pparg*, *Cidea*, *Cidec*, *Fabp4*, *Plin4*, *Cfd* and *Adig* (**Fig 4**). According to the results, the U1 samples showed the most significant changes in the expression levels of this gene set among the four genotypes. Yet, the changes were opposite between the two sexes. In the case of females, the expression levels of two genes, *Pparg* and *Fabp4*, were both 1.5-fold up-regulated compared to those of the wild type (*p* = 0.0004 for *Pparg* and *p* = 0.0025 for *Fabp4*, Mann-Whitney U test). In the case of males, the U1 samples showed significant down-regulation of all the genes for lipid metabolism, ranging from 3% to 47% levels of the WT sample (*p* = 0.0004 for all of the genes, Mann-Whitney U test). On the other hand, the other two groups, KO2 and U1/KO2, showed no major change with similar levels to those observed from WT. Besides the U1 samples, we also observed consistent down-regulation of this gene set from the female samples of the U1/KO2 genotype (*p* = 0.072 for *Pparg* and *p* = 0.0004 for the remainders, Mann-Whitney U test). This change ranged from 26 to 87% level of the WT sample (purple bars), suggesting potential effects by switching the active alleles of the *Peg3* domain.

**Fig 5:**
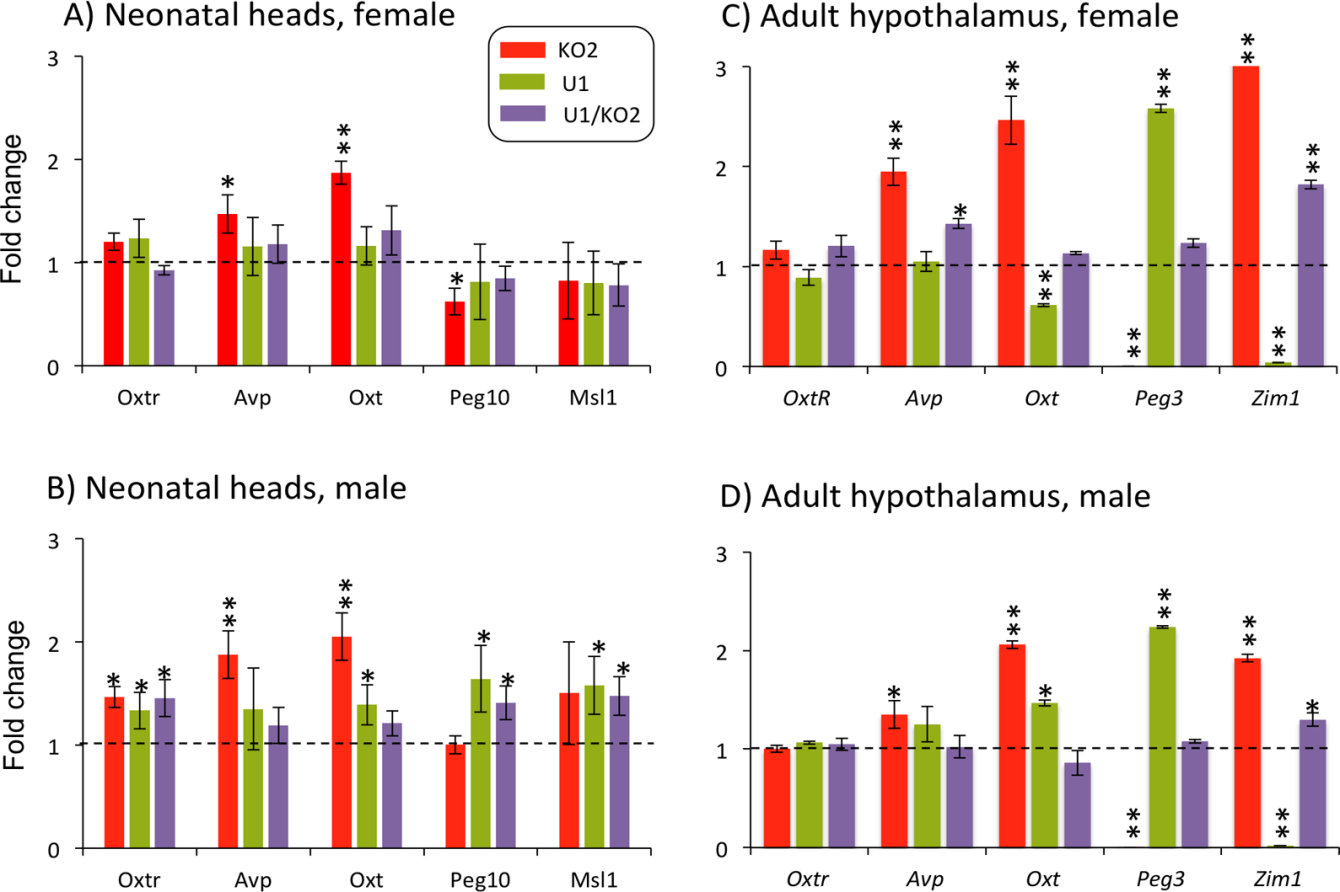
Expression levels of the *Peg3*-associated genes among the four genotypes. A series of qRT-PCR-based analyses were performed using the total RNA isolated from the neonatal heads of females (**A**) and males (**B**), and also from the adult hypothalamus of females (**C**) and males (**D**) of the four genotypes. The expression levels of each gene were first normalized with an internal control (β-actin). The subsequent value was further compared to that of the WT sample, which has been indicated with dotted lines. The relative levels were presented with standards deviation. Statistically significant differences in expression have been indicated with * being less than 0.05 and ** being less than 0.001. The corresponding p values were also pointed out in the text. This set of analyses was repeated using three biological replicates for the neonatal set and two biological replicates for the adult hypothalamus.

We also performed a similar series of expression analyses for the following genes: *Oxtr*, *Avp*, *Oxt*, *Peg10* and *Msl1* (**Fig 5**). The three genes (*Oxtr*, *Oxt* and *Avp*) and *Peg3* have been shown to be involved in maternal-caring behaviors and controlling the volume of bodily fluids [23, 24], while *Msl1* and *Peg10* are known to be downstream targets of *Peg3* [25–27]. For this series of expression analyses, we used male (n = 3) and female (n = 3) cDNA sets that had been generated using the total RNA from neonatal heads and adult hypothalamus. According to the results, the expression levels of these genes were overall similar among the four genotypes except that the levels of *Oxt* were 2.0-fold up-regulated in both males and females of the KO2 samples (*p* = 0.0004 Mann-Whitney U test) (**Fig 5A**). This initial observation was further supported by the results from the cDNA of adult hypothalamus, showing 2 to 2.5-fold up-regulation of *Oxt* in both male and female samples (*p* = 0.0004 for male and *p* = 0.0005 for female, Mann-Whitney U test) (**Fig 5C and Fig 5D**). Besides *Oxt*, we also observed up-regulation of *Avp* in adult females (*p* = 0.0004, Mann-Whitney U test) and males (0.0023 Mann-Whitney U test). Overall, this series of analyses concluded that the gene set for lipid metabolism was up and down-regulated in the female and male of the U1 genotype, respectively, and also that the expression levels of *Oxt* were up-regulated in the KO2 sample.

### Spatial expression patterns of *Peg3* and *Oxt* in the four genotypes

**Fig 6:**
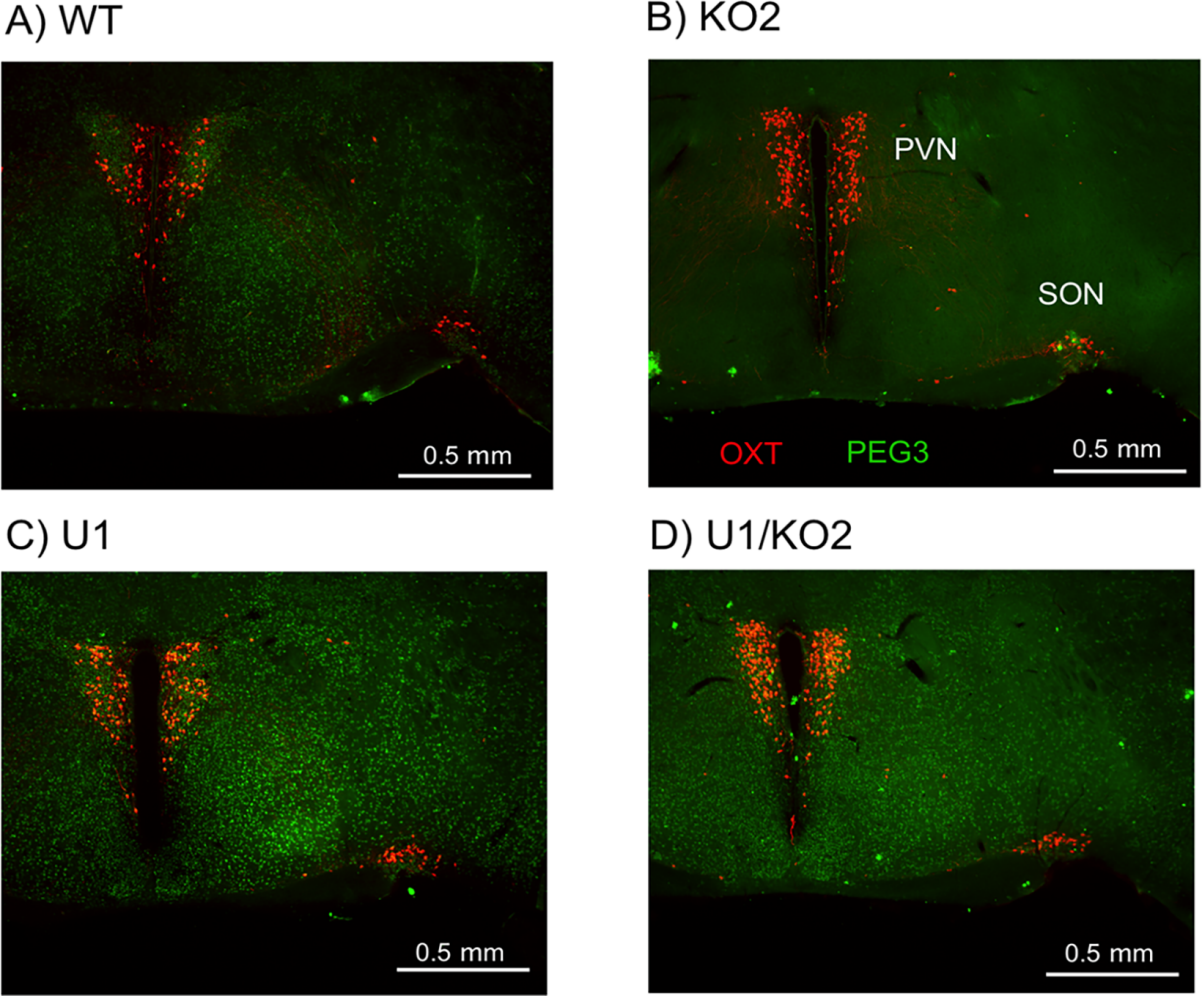
Spatial expression patterns of *Peg3* and *Oxt* within the adult hypothalamus. Double staining of PEG3 (green) and OXT (red) immunoreactive neurons in the hypothalamus of 3-month-old mice of the four genotypes (**A-D**). PEG3 immunoreactive neuronal cells were detected throughout the entire area of the hypothalamus of the three genotypes (WT, U1, U1/KO2), whereas OXT immunoreactive neurons were mainly detected from the PVN (Paraventicular nucleus) and SON (Supraoptic nucleus) areas of the hypothalamus.

We performed a series of immunostaining to monitor the spatial expression pattern of *Peg3* and also to further confirm the observed up-regulation of *Oxt* (**Fig 6**). For this series of immunostaining, we harvested a set of 8 hypothalamus samples from 3-month-old adult mice, representing the four genotypes of both sexes. This set of hypothalamus samples were sectioned to derive coronal slices with 40 μm thickness, which were subsequently used for the double staining with two antibodies: anti-PEG3 and anti-OXT antibodies. The results are summarized as follows. First, we observed the immunoreactivity with anti-PEG3 antibody from the sectioned slices of the three genotypes, WT, U1, U1/KO2 (green dots), but not from those of the KO2 genotype. This agrees with the expression of *Peg3* within the hypothalamus of the three genotypes, WT, U1, U1/KO2, and also with no expression of *Peg3* in the KO2 sample (**Fig 5C and Fig 5D**). The signals were somewhat stronger in the U1 sample than the other two genotypes, WT and U1/KO2. This is also consistent with the fact that the expression levels in the U1 were 2-fold higher than those from the other two genotypes (**Fig 5C and Fig 5D**). Second, we observed the immunoreactivity with anti-OXT antibody from the four genotypes, mainly from the PVN (Paraventricular nucleus) and SON (Supraoptic nucleus) areas (red dots in **Fig 6**). We further counted the number of neuronal cells that were immunoreactive with the anti-OXT antibody using the entire set of sectioned slices, about 70 slices per each genotype. The total numbers of the positive neuronal cells were 2376 for WT, 3217 for KO2, 2794 for U1, 3627 for U1/KO2. The relatively high number from the KO2 sample also agrees with the up-regulation of *Oxt* observed from KO2 through qRT-PCR analyses (**Fig 5C and Fig 5D**). The observations described above were derived from the male set of the hypothalamus. Overall, this series of immunostaining confirmed the similar spatial expression pattern of *Peg3* among the three genotypes, WT, U1, U1/KO2, and also detailed the up-regulation of *Oxt* in the adult hypothalamus of the KO2 genotype.

### Survival and growth rates of the four genotypes

**Fig 7:**
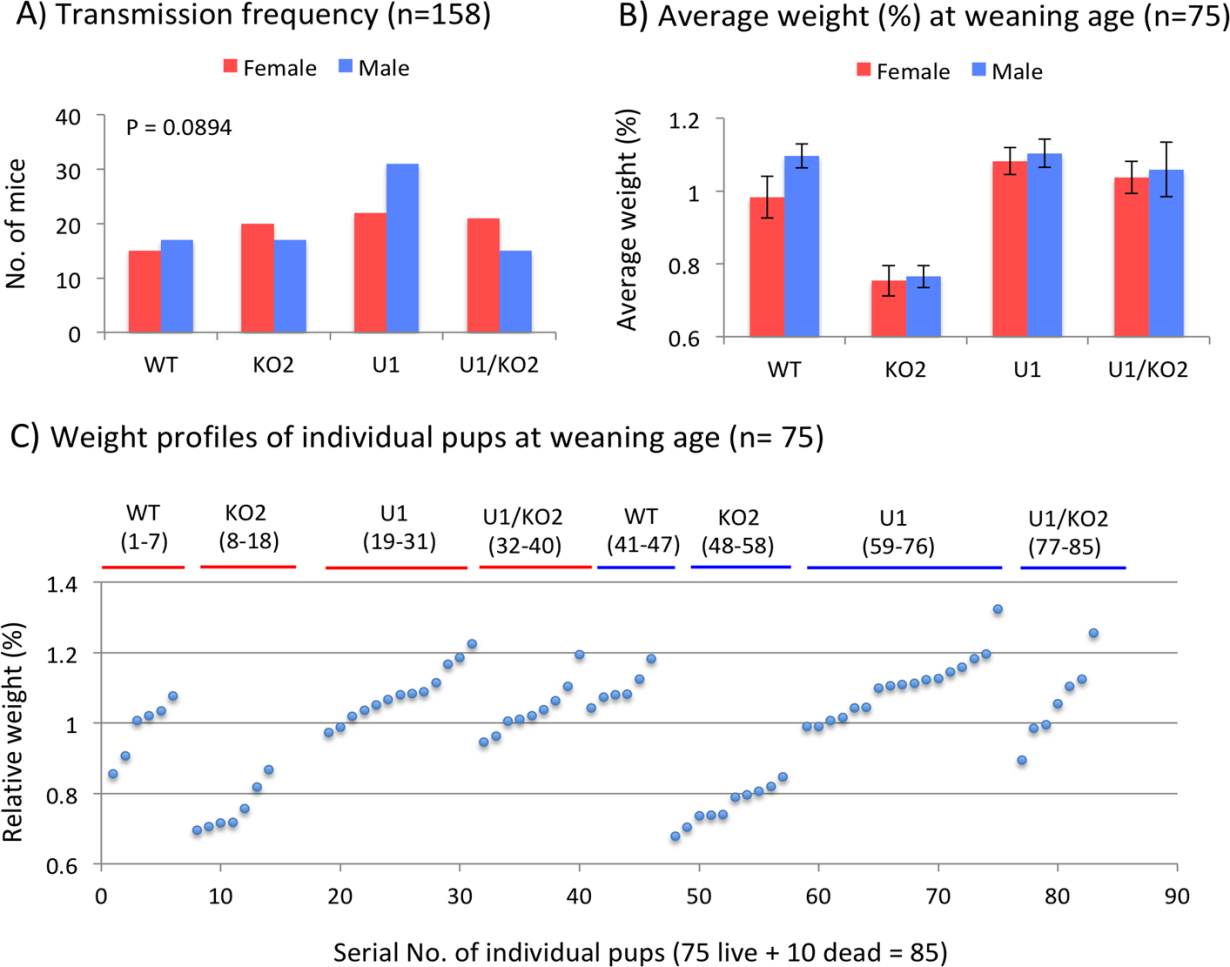
Summary of breeding experiments. A total of 158 pups derived from the breeding were summarized based on the transmission frequency of each mutant allele (**A**), the average weight (**B**), and also the weight profile of individual pups (**C**). Each pup was measured at the birth and weaning ages, which was then further divided with the average weight of the litter. The subsequent value (%) was used for calculating the average weight for each genotype (**B**) and also the individual weight profile (**C**).

We also analyzed the generated pups in terms of their survival and growth rates (**Fig 7**). For this series of analyses, the pups were sexed and genotyped, and also their weights were measured at either birth (postnatal day 1) or weaning ages (postnatal day 21). The results are summarized as follows. First, the intercrossing between two heterozygotes, females with the maternal deletion of the U1 promoter and males with the paternal deletion of the Peg3-DMR (**Fig 2A**), derived a total of 158 pups for 18 litters with the average litter size being 8.72. This litter size is considered to be normal for the mouse of the C57BL/6J background, thus no embryonic lethality is thought to be associated with this breeding scheme, especially after the implantation stage. Out of the 158 pups, 13 pups died within a couple of days after their birth, but with no clear association to any genotype (**S1 File**). These 158 pups were further divided into the four genotypes in the following manner: 32 pups (15 Females + 17 Males) for WT; 37 pups (20 Females + 17 Males) for KO2; 53 pups (22 Females + 31 Males) for U1; and 36 pups (21 Females + 15 Males) for U1/KO2 (**Fig 7A**). Given the observed frequencies, the U1 genotype appeared to have the highest number of pups among the four genotypes, although statistically not significant (p = 0.08, chi-square test). Thus, this suggests that the double gene dosage of *Peg3* and *Usp29* might provide U1 mutants a competitive advantage. It is also interesting to note that males tend to be over-represented in the U1 group (22 Females + 31 Males), which is consistent with the previous study that females were under-represented among the mutants with the double dosage of *Peg3* and *Usp29* [20]. Second, the weight profiles revealed that the KO2 genotype showed significant reduction (*p* < 0.0001, student’s t-test) in their body weights in both sexes (**Fig 7B and Fig 7C and S1 File)**, which agrees with the previous studies [12–15]. The weight profiles also revealed two unique observations. In the case of the U1 genotype, the double dosage of *Peg3* and *Usp29* seemed to have a major effect on the growth rate of the female pups based on the average weight difference between U1 (108%) and WT (98%) (*p* = 0.004, student’s t-test). This difference, however, was not detected in the males (110% in both U1 and WT genotypes, *p* = 0.42, student’s t-test). Thus, the double dosage of *Peg3* and *Usp29* and/or reduction of *Zim1* appeared to result in increased growth rates only in the female pups. In the case of the U1/KO2 genotype, the weight of females became heavier than that of WT (103% versus 98%), whereas the weight of the males became lighter than that of WT (105% versus 110%). However, neither were statistically significant from WT (*p* = 0.077 for females and *p* = 0.2 for males, student’s t-test). In conclusion, this series of breeding experiments provided two main observations: 1) the U1 genotype may have a competitive advantage and 2) the growth rate of KO2 mice was drastically reduced while the growth rate of U1 female mice was increased.

## Discussion

In the current study, we characterized the allele and dosage specificity associated with the *Peg3* domain by using two mutant alleles targeting the Peg3-DMR and the alternative U1 promoter. According to the results, the mutants with abrogation of *Peg3* and *Usp29* expression showed drastic reductions in growth rates for both male and female mice. In contrast, the mutant group with double dosage of *Peg3* and *Usp29* displayed an increase in growth rate of female mice. The mutant with switched active alleles did not cause any major impact on animal growth and survival, but displayed some differences in gene expression compared to wild type. Overall, the gene dosage of the *Peg3* domain is closely associated with the growth rates of the animals.

The mutant with double dosage of *Peg3* and *Usp29* appeared to be the fittest among the four genotypes based on the highest survival rates and growth rates (**Fig 7**). Nevertheless, the *Peg3* domain has been maintained as a single-dosage imprinted domain throughout mammalian evolution [11], suggesting the presence of some unknown functional constraints hindering the evolutionary selection of the animals with double dosage of *Peg3* and *Usp29*. The *Peg3* domain has been shown to be sexually biased according to previous studies [17, 28, 29]. The expression levels of the *Peg3* domain are higher in males than in females, and also mutations on this locus tend to have different effects between two sexes. The current study also provides a similar observation that the mutant group with the double dosage of *Peg3* and *Usp29* was somewhat over-represented by males (**Fig 7A**), which is consistent with the results from the previous study [20]. Thus, it is feasible to predict that different optimum gene dosages between two sexes might be a potential constraint maintaining the imprinting of the *Peg3* domain, which might be interesting to pursue in the near future.

The gene dosage of the *Peg3* domain appeared to be closely associated with the expression levels of the gene set involved in lipid metabolism (**Fig 4**). This is also consistent with the results from the previous studies demonstrating that loss-of-function type mutations in *Peg3* tend to result in smaller body sizes but with higher percentages of adipocytes, and also that lipid metabolism is one of the biological pathways that are affected in the mutants targeting *Peg3* [13, 14]. According to the results from the current study, however, the increased dosage of *Peg3*/*Usp29* resulted in two opposite outcomes between sexes: the gene set for lipid metabolism was significantly down-regulated in males whereas up-regulated in females (**Fig 4**). This is further supported by the female mutants with double dosage of *Peg3*/*Usp29*, which showed the highest growth rates among the four genotypes (**Fig 7**). The increased growth rates were also more pronounced prior to weaning, when the main diet is fat-rich milk for the pups. During the postnatal stage, the milk may be converted into the fat depots of this mutant more efficiently than the other three genotypes, since the females of this mutant have already elevated expression levels of the gene set for lipid metabolism (**Fig 4**). As an imprinted gene, Peg3's close association with lipid metabolism also agrees with the idea that genomic imprinting may have been implemented to provide placental mammals the genetic means by which females can provide fat-rich milk to the offspring [4–7]. Overall, lipid metabolism appears to be one of the most responsive pathways that are affected by the increased gene dosage of the *Peg3* domain.

The mutant with switched active alleles (U1/KO2) displayed overall similar but slightly different phenotypic outcomes from the wild type. The observed differences include slightly different expression levels of the *Peg3* domain and different body weights compared to the wild type (**Fig 4 and Fig 7**). These observed differences might be an indication that the two alleles of the *Peg3* domain may still be different from each other regardless allelic-specific DNA methylation on the Peg3-DMR. One feasible explanation would be that some unknown enhancers might be allele-specific. In that regard, it is relevant to note that the *Peg3* domain contains the 200-kb genomic region that harbors about 20 evolutionarily conserved regions (ECRs) [30, 31]. Some of these putative enhancers might be paternal-specific and responsible for the spatial and temporal expression patterns of the paternally expressed *Peg3* and *Usp29*. The other group of enhancers might be maternal-specific and control the expression pattern of the maternally expressed *Zim1*. In this situation, switching alleles might put the promoters of individual genes under a different set of enhancers, causing slightly different expression levels and patterns for the associated imprinted genes. If this is indeed the case, this would have the following implications. First, genomic imprinting may be regulated not only through ICRs but also through allele-specific enhancers. Second, it is likely that the reactivated imprinted allele for a given domain may not be biologically equivalent to the opposite active allele, which normally produces functional gene products. This further implicates that reactivating the repressed alleles for human imprinting disorders, such as Prader-Willi/Angelman syndromes, may not compensate completely for the loss of the corresponding active alleles [32, 33]. Overall, investigating this possibility in the near future should be of great interest given its scientific and clinical significances.

## Material and methods

### Ethics statement

All the experiments related to mice were performed in accordance with National Institutes of Health guidelines for care and use of animals, and also approved by the Louisiana State University Institutional Animal Care and Use Committee (IACUC), protocol #16–060.

### Mouse breeding

In the current study, we used two mutant strains that have been previously characterized: the KO2 strain with the 4-kb deletion of the Peg3-DMR and the U1ΔR strain with the 1-kb deletion of the alternative U1 promoter [17, 20]. Ten female heterozygotes for the U1ΔR allele were crossed with five male heterozygotes for the KO2 allele. The subsequent pups were analyzed in terms of sex, genotype and weight. Statistical significance of potential difference in the sex ratio, allele frequency and average weight between the genotypes was assessed using chi-squared test. For genotyping, genomic DNA was isolated from either clipped ears or tail snips by incubating the tissues overnight at 55°C in the lysis buffer (0.1 M Tris-Cl, pH 8.8, 5 mM EDTA, pH 8.0, 0.2% SDS, 0.2 M NaCl, 20 μg/ml Proteinase K). The isolated DNA was subsequently genotyped using the following two sets of primers: for the KO2 allele, Primer A (5’-TGACAAGTGGGCTTGCTGCAG-3’), B (5’-GGATGTAAGATGGAGGCACTGT-3’), and D (5’-AGGGGAGAACAGACTACAGA-3’); for the U1ΔR allele, P1 (5’-TAGCAAGGGAGAGGGCCTAG-3’), P2 (5’-GGAAGCCTCCATCCGTTTGT-3’), and P3 (5’-AGCACAGCTAGAAATACACAGA-3’). The sex of the pups was determined through PCR using the following primer set: mSry-F (5’-GTCCCGTGGTGAGAGGCACAAG-3’) and mSry-R (5’-GCAGCTCTACTCCAGTCTTGCC-3’).

### DNA methylation analysis

For DNA methylation analyses, the genomic DNA isolated from tail snips were treated with the bisulfite conversion reaction according to the manufacturer’s protocol (EZ DNA methylation kit, Zymo Research) [21]. The converted DNA was used as a template for the PCR reaction using the primer set that was designed for amplifying each target region. The amplified products were analyzed with COBRA (COmbined Bisulfite and Restriction Analysis) [22]. The information regarding the sequences of oligonucleotides and the PCR conditions for each genomic region is also available through the previous study [17].

### Expression analyses

Total RNA was isolated from the tissues of one-day-old heads or the hypothalamus of adult mice using a commercial kit (Trizol, Invitrogen). The total RNA was then reverse-transcribed using the M-MuLV kit (Invitrogen), and the subsequent cDNA was used as a template for quantitative real-time PCR. This analysis was performed with the iQ SYBR green supermix (Bio-Rad) using the ViiA*™* 7 Real-Time PCR System (Life Technologies). All qRT-PCR reactions were carried out for 40 cycles under standard PCR conditions. The analyses of the results from qRT-PCR were described previously [34]. Statistical significance of potential difference of expression levels of a given gene between two samples was tested with Mann-Whitney U test. The information regarding individual primer sequences is available through the previous study [14, 17].

### Immunohistochemistry

Each mouse was anesthetized with an intraperitoneal injection of a ketamine/xylazine cocktail (87.5 mg/kg ketamine; 12.5 mg/kg xylazine) at a dosage of 0.1 ml per 20-gram body weight. The animals were then transcardially perfused with 0.1 M sodium phosphate-buffered saline (PBS: pH 7.2–7.4) and fixed with 4% paraformaldehyde in 0.1 M phosphate buffer (PB: pH 7.2–7.4). Mice were decapitated, and heads were post-fixed overnight in the same fixative. Brains were dissected and blocked on either side of the hypothalamus. Coronal sections were transected from the hypothalamus at 40 μm thickness using a vibratome (Leica VT1200 S, Leica, Mannheim, Germany), and placed in PBS containing 0.5% Triton X‐100 (PBST). The free‐floating brain sections were incubated with an in-house primary antibody against PEG3 [14] and the primary PS38 antibody against oxytocin-neurophysin (provided by H. Gainer, NIH) at dilutions of 1:1000 and 1:500, respectively. This incubation was carried out in PBST for 48–72 hours at 4°C with continuous gentle agitation. Sections were washed three times with fresh PBST, followed by incubation with goat anti-rabbit antibody conjugated with DyLight 488 (Jackson ImmunoResearch, West Grove, PA) and DyLight 649 (Jackson ImmunoResearch, West Grove, PA). Both incubations were performed at 1:400 dilution in PBST overnight. The sections were washed three times with PBST, mounted on gelatin-coated slides, dehydrated, cleared, and cover-slipped with an anti-fading agent that consists of 4.8g PVA, 12g glycerol, 12 mL dH_2_O, 24 mL 0.2M Tris-HCl, and 1.25g DABCO (1,4-diazabicyclo[2.2.2]octane). Fluorescence images were acquired digitally (Eclipse 80i equipped with a digital camera, DS‐QiMc, Nikon, Tokyo, Japan). ImageJ software was used to process the images in dynamic range with minimal alterations.

## Supporting information

S1 File This file contains the raw data from the breeding experiment, including transmission frequencies of each mutant group and weight data of individual mice, which have been summarized as Fig 7. (XLSX)
